# Psychological outcomes and discharge readiness of parents of preterm infants in China after family integrated care

**DOI:** 10.3389/fped.2026.1653759

**Published:** 2026-02-09

**Authors:** Yuanyuan Zhu, Yaqiong Jiang, Fei Hong, Lei Song, Xiaohong Yang

**Affiliations:** Department of Neonatology, Nantong First People's Hospital, Nantong, Jiangsu, China

**Keywords:** discharge readiness, family integrated care, NICU, parental psychological outcomes, premature infants

## Abstract

**Objective:**

This study aimed to evaluate the effects of Family Integrated Care (FICare) on the psychological outcomes and discharge readiness of parents of premature infants in the neonatal intensive care unit (NICU).

**Methods:**

This prospective, non-randomized controlled study was conducted in the NICU of Nantong First People's Hospital (China). Parental psychological outcomes (trauma, depression, anxiety, and stress) were assessed using the Trauma Screening Questionnaire (TSQ), post-traumatic stress disorder (PTSD) Checklist for DSM-5 (PCL-5), and the Depression Anxiety Stress Scales (DASS-21) at two timepoints: within two weeks postpartum and at three months after discharge. Discharge readiness was evaluated using validated maternal and paternal readiness scales. Infant clinical outcomes, including nutritional and respiratory milestones, were also recorded. Statistical analysis was conducted using SPSS 23.0 software, and statistical significance was set at *p* < 0.05.

**Results:**

A total of 84 families with preterm infants born at 28–34 weeks of gestation were enrolled and allocated into either the FICare group (*n* = 42) or the standard care group (*n* = 42). Baseline demographic and clinical characteristics were comparable between the two groups. FICare significantly improved discharge readiness in both mothers and fathers at NICU discharge (*p* < 0.001 for all subscales). Parental psychological outcomes in the FICare group showed significant improvements at three months after discharge, including reduced rates of clinically significant trauma (TSQ-positive: mothers 2.38% vs. 21.43%, fathers 4.76% vs. 26.19%; all *p* < 0.05) and PTSD (PCL-5-positive: mothers 2.38% vs. 23.81%, fathers 2.38% vs. 19.05%), along with decreased symptoms of anxiety, depression, and stress (all *p* < 0.05). In terms of infant outcomes, the FICare group exhibited earlier initiation of enteral feeding (2.14 ± 0.95 vs. 3.45 ± 0.94 days, *p* < 0.001), quicker achievement of full enteral feeding (15.81 ± 4.62 vs. 20.45 ± 3.70 days, *p* < 0.001), and higher discharge weight (2,202.07 ± 167.40 g vs. 1,982.94 ± 176.31 g, *p* < 0.001), all without prolonging NICU stay or respiratory support.

**Conclusion:**

FICare significantly enhanced the psychological well-being and discharge preparedness of parents of preterm infants, while also promoting improved nutritional outcomes in neonates.

## 1 Introduction

Preterm birth, defined as delivery before 37 completed weeks of gestation, accounts for approximately 11% of all live births worldwide and remains a leading cause of neonatal morbidity and mortality (1). Despite significant advancements in neonatal intensive care, which have markedly improved the survival rates of preterm infants, the consequences of prematurity mainly extend well beyond the neonatal intensive care unit (NICU). These infants are at the elevated risk of a broad range of complications, including respiratory distress (2), feeding difficulties (3), neurodevelopmental delay (4), and growth restrictions (5). Moreover, parents, particularly first-time caregivers, undergo notable psychological strain during their infant's prolonged hospitalization (6).

The NICU's environment, characterized by high-intensity medical interventions, unfamiliar technologies, and limited opportunities for parental involvement, is inherently stressful. Studies have consistently shown that parents of preterm infants frequently experience elevated levels of anxiety, depression, and post-traumatic stress symptoms (7, 8). These psychological burdens may not only impair parental mental health, but also hinder the development of effective parent-infant attachment and reduce caregiving confidence, ultimately influencing the infant's developmental trajectory post-discharge. Fathers, historically underrepresented in neonatal research, are increasingly recognized as being equally vulnerable to emotional distress. However, they mainly receive inadequate support and are frequently excluded from neonatal care processes (9).

Traditional NICU-based models predominantly concentrate on healthcare professionals delivering care while parents’ passive, observational roles are noteworthy (10). In these models, parental participation is often restricted to minimal caregiving tasks, such as diaper changing or limited breastfeeding, typically during the final days prior to discharge (10). This limited engagement contributes to parental feelings of helplessness and unpreparedness, and it may lead to reduced caregiving competence at home, increased risk of readmission, and delayed recovery for the infant. To address these limitations, Family Integrated Care (FICare) has emerged as an innovative and family-centered model of NICU care (10–12). Originating in Canada and increasingly implemented globally, FICare was designed to empower parents through structured education, hands-on training, and active participation in the care of their hospitalized infants (10–12). Under FICare, parents are integrated into the care team, participating in both routine and specialized care activities under the supervision of trained NICU staff. The model also incorporates peer support, emotional counseling, and ongoing lactation guidance, thereby promoting parental self-efficacy, improving parent-infant bonding, and potentially enhancing neonatal outcomes.

Evidence from previous research demonstrated that FICare is associated with improved neonatal parameters, including shorter time to full enteral feeding, greater weight gain, higher exclusive breastfeeding rates, and reduced length of NICU stay (11, 13). Additionally, preliminary studies have reported that FICare may ameliorate maternal stress and depressive symptoms during hospitalization (14). However, despite these promising findings, substantial gaps remain in the literature. Specifically, few studies have rigorously assessed the comprehensive psychological outcomes, including trauma, anxiety, depression, and stress, in both parents using standardized measures across multiple timepoints. Furthermore, discharge readiness, a critical determinant of post-discharge success and caregiver competence, has been underexplored in association with family-centered interventions. Most existing studies have also emphasized maternal outcomes, often neglecting the equally important paternal experience.

Hence, the present prospective controlled study was conducted at the NICU of Nantong First People's Hospital, a tertiary care institution located in Nantong, China. This hospital serves a diverse patient population, including families from both urban and rural areas, representing a broad spectrum of socioeconomic backgrounds. The study aimed to evaluate the effects of a structured FICare program on parental psychological outcomes and discharge readiness in a cohort of 84 families with preterm infants admitted to the NICU.

## 2 Methods

### 2.1 Participants' enrollment

This prospective, non-randomized controlled study was conducted in the NICU of Nantong First People's Hospital from May 2024 to May 2025. Inclusion criteria were as follows: (1) infants born at 28–34 weeks' gestation; (2) both parents being first-time caregivers; (3) parental consent and ability to communicate; (4) maternal breast milk production; (5) anticipated NICU stay ≥7 days; (6) partial participation in discharge education; (7) involvement of only singleton infants in the study, ensuring consistency and homogeneity in the sample. Exclusion criteria included: (1) infant surgery; (2) in-hospital death; (3) transfer to another facility; and (4) inability of either parent to complete all study procedures. Eligible families were identified within 72 h of admission and enrolled following written informed consent. The study was approved by the Institutional Review Board of Nantong First People's hospital (Approval No. 2023KT142), and conducted in accordance with the Declaration of Helsinki.

### 2.2 Study design

Each study unit included a preterm infant and both parents. Due to logistical factors, such as shared accommodations (e.g., maternal hotel), full blinding was infeasible, and some intergroup contamination may have occurred. To reduce this risk, data collection was conducted sequentially, with one group completing participation before the other began. The order of group assignment was determined through a sealed-envelope draw carried out by an uninvolved NICU nurse, which resulted in the control group being enrolled first, followed by the FICare group. Primary outcomes included parental psychological status, assessed using validated tools for trauma, depression, anxiety, and stress at two timepoints: within the first two weeks postpartum and at three months after discharge. Discharge readiness was measured within 48 h after birth, when parents were clinically stable and communicative, and again within 24 h prior to NICU discharge. Secondary outcomes included infant clinical parameters, such as length of NICU stay, respiratory support duration, feeding milestones, and discharge weight.

### 2.3 Parental education and care procedures

#### 2.3.1 FICare procedures

All components of the FICare model were implemented progressively. Parents in the intervention group attended in a structured one-week training, limited to four couples per session, with weekend options for those unavailable on weekdays. The program covered infant care topics, such as hygiene (skin, ears, eyes, nose, mouth, diapering), breastfeeding, safe sleep, vaccination follow-up, bathing, kangaroo care, nail care, breast milk benefits, medication use, emergency response, and medical check-ups. Parents initially observed demonstrations on a model infant, followed by supervised practice. One-on-one support sessions were available on Mondays for further clarification and guidance. NICU staff received standardized training, in which nurses completed a 4-h session, and other personnel received about 3 h of instruction, all coordinated by a senior NICU nurse. After training, parents gradually participated in infant care responsibilities under the supervision of a nurse. Once clinical stability was achieved, parents were engaged in at least three sessions of caregiving and stayed in the hospital for 6–8 h daily. Before discharge, peer support was provided by families with previous NICU experience. A summary guide was provided at discharge, and lactation support, including hand expression, was initiated within 6 h postpartum, with continued breastfeeding guidance from a lactation consultant.

#### 2.3.2 Standard care procedures

In the control group, routine nursing care was provided from NICU admission to discharge. Care interactions were primarily between the nurse and the mother, who allowed to perform basic tasks, such as diapering and breastfeeding, depending on the nurse's judgment. Mothers typically began their overnight hospital stays approximately two days before discharge. Fathers were informed about the care process and involved in emotional support and communication with healthcare providers, while they did not participate directly in neonatal care tasks. This approach was consistent with the hospital's FICare protocol, primarily designating mothers as the primary caregivers during hospitalization.

### 2.4 Psychological, clinical, and readiness assessments

Parental psychological outcomes, discharge readiness, and infant clinical indicators were assessed using standardized, validated instruments at designated timepoints during the NICU stay and throughout post-discharge follow-up.

#### 2.4.1 Psychological outcomes

Parental psychological status was measured using the following instruments: (1) Trauma Screening Questionnaire (TSQ) (15): A 10-item self-report tool used to screen for post-traumatic stress symptoms. A score of ≥6 indicated a positive screen for clinically significant trauma symptoms. (2) Posttraumatic Stress Disorder Checklist for DSM-5 (PCL-5) (16): A 20-item scale assessing the severity of post-traumatic stress disorder (PTSD) symptoms based on DSM-5 criteria. A total score of ≥33 was used to indicate probable PTSD. (3) Depression Anxiety Stress Scales (DASS-21) (17): A 21-item instrument that yields subscale scores for depression, anxiety, and stress. Subscale scores were interpreted using established severity thresholds (normal to extremely severe). Psychological assessments were administered to both mothers and fathers at two timepoints: (1) within the first two weeks after birth (baseline) and (2) three months postpartum (follow-up). All research materials, including the questionnaires and assessments, were provided in Chinese to ensure that participants, all of whom were Chinese-speaking, could fully understand the content and respond accurately.

#### 2.4.2 Discharge readiness

Parental discharge readiness was assessed using two validated scales developed by Tiryaki and Çınar (18). The Maternal Readiness for Discharge and Home Care Scale consists of 22 items across four subscales, including feeding, perception of general condition, hygienic care, and care practices, rated on a 7-point Likert scale, yielding total scores from 22 to 154. The Paternal Readiness for Discharge and Home Care Scale includes 20 items, covering hygienic care, feeding and care support, and care practices, with scores ranging from 20 to 144. Higher scores of both scales indicate greater discharge readiness. Assessments were conducted twice: within 48 h postpartum when parents were communicative and stable, and again within 24 h before NICU discharge.

#### 2.4.3 Infant clinical outcomes

Clinical parameters for preterm infants were extracted from electronic medical records and included duration of NICU stay, duration of mechanical ventilation and continuous positive airway pressure (CPAP) support, time to the first breast milk intake and the first breastfeeding, time to initiation and achievement of full enteral feeding, and discharge weight.

All instruments were administered by trained researchers in a quiet, private setting. Questionnaires were completed independently by each parent. Data integrity was ensured through double data entry and periodic quality checks.

### 2.5 Sample size calculation

Although a formal *a priori* sample size calculation was not performed, a *post hoc* sample size justification was conducted based on the primary outcomes of this study. Parental discharge readiness at NICU discharge was selected as a key outcome targeted by the FICare intervention. The post-intervention maternal discharge readiness scores were 147.83 ± 8.14 in the FICare group and 119.95 ± 10.59 in the control group, yielding a mean difference of 27.88 points and an estimated large effect size (Cohen's *d* = 2.95). At a two-sided significance level of 0.05% and 90% power, fewer than 20 participants per group would have been required to detect this difference. Consistent findings were observed for neonatal nutritional outcomes, such as time to full enteral feeding (Cohen's *d* ≈ 1.10), which would require approximately 18–22 infants per group to achieve 80%–90% power. Large between-group differences were also observed for parental psychological outcomes at follow-up, further supporting the adequacy of the sample size. Accordingly, the final sample of 84 families (42 per group) provided sufficient statistical power to detect clinically meaningful differences across primary and secondary outcomes. Nevertheless, given the single-center design, future multicenter studies with formal *a priori* power calculations are warranted.

### 2.6 Statistical analysis

Data were analyzed using SPSS 23.0 software (IBM Corp., Armonk, NY, USA), and *p* < 0.05 indicated statistical significance. Descriptive statistics were used to summarize baseline characteristics and outcomes. Kolmogorov–Smirnov test was employed to determine normality of continuous variables, and mean ± standard deviation (SD) or median and interquartile range (IQR) was used to describe those with or without normal distribution. Percentages and frequencies were utilized for description of categorical variables. Between-group comparisons of continuous variables were conducted using independent-samples *t*-test for normally distributed data, and Mann–Whitney *U*-test for abnormal data. Categorical variables were analyzed using the Chi-square test or the Fisher's exact test, as appropriate. Within-group comparisons (pre- vs. post-intervention) were performed using paired *t*-test for normally distributed variables and Wilcoxon signed-rank test for non-normally distributed variables. Baseline equivalence between groups was assessed prior to intervention. All statistical procedures were reviewed by a biostatistician to ensure methodological validity.

## 3 Results

### 3.1 Baseline features of parents and NICU-admitted preterm infants in the control and FICare groups

Initially, a total of 122 eligible families with preterm infants were approached for participation. Among them, 84 families met all inclusion criteria, provided informed consent, and were successfully enrolled. These 84 families were then allocated to either the FICare group (*n* = 42) or the standard care group (*n* = 42), based on parental availability and willingness to participate. The baseline features of parents and infants in the control and FICare groups are summarized in Table 1. A total of 84 families of premature infants admitted to the NICU were enrolled, involving 42 in each group. No significant differences were found between groups in terms of maternal age (27.64 ± 1.90 vs. 28.02 ± 1.97 years, *p* = 0.369), paternal age (30.00 ± 2.08 vs. 30.64 ± 1.78 years, *p* = 0.132), or infant gestational age (31.50 ± 1.09 vs. 31.33 ± 0.93 weeks, *p* = 0.452). Maternal sociodemographic variables were comparable. Most mothers had a college-level education or above (80.95% vs. 85.71%, *p* = 0.771), were employed (83.33% vs. 88.10%), and resided in urban areas (59.52% vs. 61.90%). The two groups showed no significant differences in age at marriage (25.55 ± 1.69 vs. 24.86 ± 1.52 years, *p* = 0.052), mode of conception (92.86% vs. 97.62% spontaneous), or delivery method (19.05% vs. 33.33% cesarean, *p* = 0.214). Maternal mental health history was also comparable between the two groups. The majority had never accessed mental health services (76.19% vs. 61.90%, *p* = 0.326), and prior diagnoses (4.76% vs. 7.14%) or psychiatric admissions (14.29% vs. 9.52%) were infrequent. Most mothers were non-smokers (71.43% vs. 80.95%, *p* = 0.202). Paternal characteristics were largely equivalent. Most fathers had a college education or higher (90.48% vs. 88.10%), and their employment status was not significantly different (83.33% vs. 73.81%). Rates of recent mental health service use (9.52% vs. 19.05%), previous diagnoses (7.14% vs. 4.76%), psychiatric admissions (11.90% vs. 21.43%), and smoking status (*p* = 0.816) were comparable. Infant sex distribution (female: 56.14% vs. 47.62%, *p* = 0.512) and gestational age at birth were also similar between the two groups, confirming balanced baseline clinical profiles. Collectively, these findings indicate that the control and FICare groups were demographically and clinically comparable at baseline, ensuring the validity of subsequent outcome analyses.

**Table 1:** Baseline characteristics of parents and infants in the control and FICare groups.

Indices	Control group (*n* = 42)	FICare group (*n* = 42)	*p*
Information about the mother			
Age (mean ± SD)	27.64 ± 1.90	28.02 ± 1.97	0.37
Educational background			
College and above	34 (80.95)	36 (85.71)	0.77
Compulsory education and above	8 (19.05)	6 (14.29)	
Illiterate	0	0	
Employment status			
Yes	35 (83.33)	37 (88.10)	
No	7 (16.67)	5 (11.90)	
Place of residence			
Rural	17 (40.48)	16 (38.10)	1
Urban	25 (59.52)	26 (61.90)	
Age of marriage (mean ± SD)	25.55 ± 1.69	24.86 ± 1.52	0.05
How pregnancy occurred			
Spontaneously	39 (92.86)	41 (97.62)	0.62
Through IVF or insemination	3 (7.14)	1 (2.38)	
Mode of delivery			
Normal birth	34 (80.95)	28 (66.67)	0.21
Cesarean section	8 (19.05)	14 (33.33)	
Mental health history			
Accessed mental health service in last 12 months	3 (7.14)	7 (16.67)	0.33
More than 12 months ago	7 (16.67)	9 (21.43)	
Never seen	32 (76.19)	26 (61.90)	
Previous mental health diagnosis			
Yes	2 (4.76)	3 (7.14)	1
No	40 (95.24)	39 (92.86)	
Previous admission to mental health unit			
Yes	6 (14.29)	4 (9.52)	0.74
No	36 (85.71)	38 (90.48)	
Smoke			
Current	2 (4.76)	4 (9.52)	0.20
Past	10 (23.81)	4 (9.52)	
Never	30 (71.43)	34 (80.95)	
Information about the father			
Age (mean ± SD)	30.00 ± 2.08	30.64 ± 1.78	0.13
Educational background			
College and above	38 (90.48)	37 (88.10)	1
Compulsory education and above	4 (9.52)	5 (11.90)	
Illiterate	0		
Employment status			
Yes	35 (83.33)	31 (73.81)	
No	7 (16.67)	11 (26.19)	
Mental health history			
Accessed mental health service in last 12 months	4 (9.52)	8 (19.05)	
More than 12 months ago	0	2 (4.76)	
Never seen	38 (90.48)	32 (76.19)	
Previous mental health diagnosis			
Yes	3 (7.14)	2 (4.76)	1
No	39 (92.86)	40 (95.24)	
Previous admission to mental health unit			
Yes	5 (11.90)	9 (21.43)	0.40
No	37 (88.10)	33 (78.57)	
Smoke			
Current	10 (23.81)	8 (19.05)	0.82
Past	13 (30.95)	15 (35.71)	
Never	19 (45.24)	19 (45.24)	
Information about the infant			
Infant's sex			
Female	24 (56.14)	20 (47.62)	0.51
Male	18 (42.86)	22 (52.38)	
Week of gestation (mean ± SD)	31.50 ± 1.09	31.33 ± 0.93	0.452

SD, standard deviation; IVF, *in vitro* fertilization; mo, months.

### 3.2 Differences in clinical outcomes between infants in the control group and those in the FICare group

The clinical outcomes of NICU-admitted preterm infants in the control and FICare groups are summarized in Table 2. A total of 84 infants were included, involving 42 in each group. No significant differences were found in the length of NICU stay (28.38 ± 5.63 vs. 27.29 ± 5.06 days, *p* = 0.351), days on mechanical ventilation (2.55 ± 1.21 vs. 2.69 ± 1.52, *p* = 0.636), or CPAP support (3.36 ± 1.76 vs. 3.74 ± 1.56 days, *p* = 0.298), indicating comparable respiratory needs and hospitalization time. In contrast, nutritional outcomes were more notable in the FICare group. Discharge weight was significantly higher (2,202.07 ± 167.40 vs. 1,982.94 ± 176.31 g, *p* < 0.001), and enteral feeding milestones were achieved earlier, including initiation (2.14 ± 0.95 vs. 3.45 ± 0.94 days, *p* < 0.001) and full enteral feeding (15.81 ± 4.62 vs. 20.45 ± 3.70 days, *p* < 0.001). The time to the first breast milk intake (2.40 ± 1.40 vs. 4.67 ± 1.46 days, *p* < 0.001) and the first breastfeeding (12.67 ± 3.24 vs. 17.76 ± 3.51 days, *p* < 0.001) was also significantly shorter in the FICare group. These results indicated that FICare was associated with earlier feeding progression and improved growth in preterm infants, without prolonging respiratory support or NICU stay, potentially reflecting the benefits of enhanced parental involvement.

**Table 2:** Comparison of clinical outcomes of premature infants in the NICU between the control group and the FICare group.

Variables	Control (*n* = 42)	FICare group (*n* = 42)	*p*
Discharge weight	1,982.94 ± 176.31	2,202.07 ± 167.40	<0.01
Number of days spent in the NICU	28.38 ± 5.63	27.29 ± 5.06	0.35
Number of MV days	2.55 ± 1.21	2.69 ± 1.52	0.64
Number of CPAP days	3.36 ± 1.76	3.74 ± 1.56	0.30
Enteral feeding start day	3.45 ± 0.94	2.14 ± 0.95	<0.01
Full enteral feeding start day	20.45 ± 3.70	15.81 ± 4.62	<0.01
Time of the first breast milk intake	4.67 ± 1.46	2.40 ± 1.40	<0.01
Time of breastfeeding for the first time	17.76 ± 3.51	12.67 ± 3.24	<0.01

NICU, neonatal intensive care unit; MV, mechanical ventilation; CPAP, continuous positive airway pressure.

### 3.3 Differences in parental discharge readiness and care competence between the control and FICare groups

The comparison of parental discharge readiness and care competence between the two groups is summarized in Table 3. At baseline (t₁), there were no significant differences in maternal scores for feeding, hygienic care, perception of general condition, care practices, or discharge readiness (all *p* > 0.05). Similarly, paternal scores in support for care, care practices, hygienic care, and discharge readiness were comparable between the two groups at t₁ (all *p* > 0.05). Following the intervention (t₂), parents in the FICare group showed significantly higher scores than controls across all domains. Mothers in the FICare group experienced greater improvements in feeding (21.33 ± 3.01 vs. 14.60 ± 3.09), hygienic care (46.62 ± 4.95 vs. 27.57 ± 4.39), perception of condition (26.36 ± 4.86 vs. 17.43 ± 4.13), care practices (55.55 ± 4.70 vs. 42.57 ± 5.58), and discharge readiness (147.83 ± 8.14 vs. 119.95 ± 10.59) (all *p* < 0.001). Fathers in the FICare group also outperformed the control group in post-intervention support for feeding and care (48.21 ± 5.17 vs. 36.64 ± 5.90), care practices (35.31 ± 3.84 vs. 27.29 ± 4.12), hygienic care (42.74 ± 4.65 vs. 23.17 ± 5.64), and discharge readiness (125.40 ± 10.00 vs. 87.52 ± 11.07) (all *p* < 0.001). Within-group comparisons confirmed significant improvements across all indicators in both groups (*p* < 0.001), with more remarkable gains found in the FICare group. These results demonstrated that FICare could significantly enhance parental caregiving competence and discharge readiness for premature infants in the NICU setting.

**Table 3:** Comparison of parental discharge readiness and care competence before and after family integrated care in the NICU.

Variables	Control (*n* = 42)	FICare group (*n* = 42)	*p* ^a^
Assessment for mother			
Feeding (t₁)	11.21 ± 1.52	10.93 ± 1.64	0.41
Feeding (t₂)	14.60 ± 3.09	21.33 ± 3.01	<0.01
P^b^	<0.01	<0.01	
Hygienic care (t₁)	23.45 ± 1.82	23.81 ± 1.95	0.39
Hygienic care (t₂)	27.57 ± 4.39	46.62 ± 4.95	<0.01
P^b^	<0.01	<0.01	
Perception of the general condition (t₁)	14.86 ± 1.95	14.71 ± 2.23	0.76
Perception of the general condition (t₂)	17.43 ± 4.13	26.36 ± 4.86	<0.01
P^b^	0.01	<0.01	
Care practices (t₁)	33.69 ± 3.33	34.33 ± 2.80	0.34
Care practices (t₂)	42.57 ± 5.58	55.55 ± 4.70	<0.01
P^b^	<0.01	<0.01	
The scale evaluating readiness of the mother for the discharge and at-home care of premature infants from the NICU (t₁)	87.74 ± 16.68	86.62 ± 14.04	0.74
The scale evaluating readiness of the mother for the discharge and at-home care of premature infants from the NICU (t₂)	119.95 ± 10.59	147.83 ± 8.14	<0.001
P^b^	<0.01	<0.01	
Assessment for father			
Support for feeding and care (t₁)	27.26 ± 4.58	25.57 ± 4.99	0.11
Support for feeding and care (t₂)	36.64 ± 5.90	48.21 ± 5.17	<0.01
P^b^	<0.001	<0.001	
Care practices (t₁)	22.19 ± 3.34	20.57 ± 4.39	0.06
Care practices (t₂)	27.29 ± 4.12	35.31 ± 3.84	<0.01
P^b^	<0.01	<0.01	
Hygienic care (t₁)	15.07 ± 2.02	15.50 ± 1.88	0.316
Hygienic care (t₂)	23.17 ± 5.64	42.74 ± 4.65	<0.001
P^b^	<0.01	<0.01	
The scale evaluating readiness of the father for the discharge and at-home care of premature infants from the NICU (t₁)	68.48 ± 6.04	66.07 ± 6.45	0.081
The scale evaluating readiness of the father for the discharge and at-home care of premature infants from the NICU (t₂)	87.52 ± 11.07	125.40 ± 10.00	<0.001
P^b^	<0.01	<0.01	

NICU, neonatal intensive care unit.

t₁: pre-intervention assessment; t₂: post-intervention assessment.

P_a_: between-group comparison (independent samples *t*-test); P_b_: within-group comparison (paired samples *t*-test).

SD, standard deviation.

*P* < 0.05 considered statistically significant.

### 3.4 Differences in psychological outcomes between parents of NICU-admitted preterm infants in the control and FICare groups

The psychological outcomes of parents in the control and FICare groups are presented in Table 4. At baseline, no significant differences were found between groups in terms of maternal or paternal trauma symptoms (TSQ and PCL-5), or in DASS-assessed depression, anxiety, and stress (all *P* > 0.05). At follow-up, significant improvements were identified in the FICare group. Among mothers, rates of TSQ- and PCL-5-positive screens were markedly lower than in the control group (2.38% vs. 21.43%, *p* = 0.015; 2.38% vs. 23.81%, *p* = 0.007, respectively). Fewer mothers in the FICare group reported symptoms of depression (*p* = 0.013), anxiety (*P* = 0.004), and stress (*p* = 0.021), with most classified as asymptomatic. Fathers in the FICare group also experienced significantly lower rates of trauma symptoms at follow-up (TSQ: 4.76% vs. 26.19%, *p* = 0.013; PCL-5: 2.38% vs. 19.05%, *p* = 0.029), as well as reduced rates of depression (*p* = 0.039), anxiety (*p* = 0.013), and stress (*p* = 0.004) symptoms. These results demonstrated that FICare could significantly improve parental psychological outcomes, particularly by alleviating trauma and emotional distress during the NICU hospitalization.

**Table 4:** Comparison of psychological outcomes of parents of premature infants in the NICU at baseline and follow-up between control and family integrated care groups.

Variables	Baseline		*p*	Follow-up		*p*
	Control (*n* = 42)	FICare group (*n* = 42)		Control (*n* = 42)	FICare group (*n* = 42)	
Information for Mother						
Trauma						
TSQ positive screen	9 (21.43)	6 (14.29)	0.57	9 (21.43)	1 (2.38)	0.02
PCL-5 positive screen	5 (11.90)	7 (16.67)	0.756	10 (23.81)	1 (2.38)	0.01
Depression (DASS)						
None	32 (76.19)	34 (80.95)	0.889	29 (69.05)	39 (92.86)	0.01
Mild-moderate	9 (21.43)	7 (16.67)		11 (26.19)	2 (4.76)	
Severe-extremely severe	1 (2.38)	1 (2.38)		2 (4.76)	1 (2.38)	
Anxiety (DASS)						
None	28 (66.67)	31 (73.81)	0.851	24 (57.14)	37 (88.10)	0.04
Mild-moderate	12 (28.57)	9 (21.43)		16 (38.10)	4 (9.52)	
Severe-extremely severe	2 (4.76)	2 (4.76)		2 (4.76)	1 (2.38)	
Stress (DASS)						
None	30 (71.43)	31 (73.81)	0.865	26 (61.91)	37 (88.10)	0.02
Mild-moderate	10 (23.81)	8 (19.05)		14 (33.33)	4 (9.52)	
Severe-extremely severe	2 (4.76)	3 (7.14)		2 (4.76)	1 (2.38)	
Information for Father						
Trauma						
TSQ positive screen	6 (14.29)	4 (9.52)	0.738	11 (26.19)	2 (4.76)	0.01
PCL-5 positive screen	4 (9.52)	6 (14.29)	0.738	8 (19.05)	1 (2.38)	0.02
Depression (DASS)						
None	34 (80.95)	33 (78.57)	0.843	30 (71.43)	39 (92.86)	0.03
Mild-moderate	5 (11.90)	7 (16.67)		10 (23.81)	2 (4.76)	
Severe-extremely severe	3 (7.14)	2 (4.76)		2 (4.76)	1 (2.38)	
Anxiety (DASS)						
None	33 (78.57)	36 (85.71)	0.771	30 (71.43)	39 (92.86)	0.013
Mild-moderate	8 (19.05)	5 (11.90)		11 (26.19)	2 (4.76)	
Severe-extremely severe	1 (2.38)	1 (2.38)		1 (2.38)	1 (2.38)	
Stress (DASS)						
None	32 (76.19)	35 (83.33)	0.673	29 (69.05)	40 (95.14)	0.004
Mild-moderate	8 (19.05)	6 (14.29)		10 (23.81)	1 (2.38)	
Severe-extremely severe	2 (4.76)	1 (2.38)		3 (7.14)	1 (2.38)	

TSQ, trauma screening questionnaire; PCL-5, PTSD checklist for DSM-5; DASS, depression anxiety stress scale; SD, standard deviation; NICU, neonatal intensive care unit.

## 4 Discussion

This prospective controlled study demonstrated that FICare could significantly improve psychological outcomes and discharge readiness among parents of preterm infants, while also promoting earlier achievement of nutritional milestones in infants without prolonging NICU stay or increasing respiratory support requirements. These findings support the growing body of evidence that structured, parent-inclusive models of NICU care yield both psychosocial and clinical benefits for families of premature infants.

A key finding of this study is the significant reduction in psychological distress, specifically trauma symptoms, anxiety, depression, and stress, in both mothers and fathers in the FICare group, as assessed at three months postpartum. Compared with parents receiving standard care, those participating in FICare had markedly lower rates of clinically significant trauma (TSQ and PCL-5 positive), and fewer exhibited moderate-to-severe symptoms on DASS-21 subscales. This result likely reflects the protective psychological effects of early and sustained parental involvement in the NICU environment. Through structured education, practical caregiving experience, and supervised participation in decision-making, FICare empowers parents, enhances their perceived control, and reduces helplessness, recognizing as factors that may mitigate stress and trauma (19, 20). In contrast, standard care, which limits parental interaction and relegates caregivers to passive roles, may exacerbate psychological distress by reinforcing feelings of exclusion and uncertainty. The psychological impact of preterm birth on parents, particularly during and after a prolonged NICU stay, has been well-documented in the literature. Numerous studies have demonstrated that parents of preterm infants are at significantly higher risk of developing PTSD, depressive symptoms, anxiety, and chronic stress compared with parents of term infants (8, 21–23). These symptoms often originate from the sudden and traumatic onset of premature birth, compounded by uncertainty about the infant's prognosis, unfamiliar medical environments, and a loss of perceived parental role (8, 21–23). Maternal mental health outcomes have traditionally received the most attention; however, emerging evidence demonstrates that fathers also experience a significant emotional burden, although it is mainly internalized or underreported (24–26). Persistent psychological distress in parents has been associated with poorer parent-infant bonding, reduced caregiving competence, and long-term adverse effects on child socio-emotional development (24–26). This study is one of the few to specifically evaluate paternal psychological outcomes, an often underrepresented aspect of NICU research. Fathers in the FICare group, similar to mothers, experienced significant mental health benefits. This may be attributed to the inclusion of both parents in training sessions and caregiving routines, providing shared understanding, emotional support, and parity in caregiving responsibilities. Comprehensive family-centered care models may play a critical role in interrupting the trajectory from NICU-related stress to long-term psychological morbidity, highlighting the importance of integrating mental health support into neonatal care practices.

Another key finding is the significantly greater discharge readiness in the FICare group, as reflected by higher scores on validated parental readiness scales. Both mothers and fathers in this group outperformed controls across all domains, including feeding, hygienic care, and perception of infant condition. These findings likely stem from the structured, hands-on FICare training, emphasizing skill-building, knowledge reinforcement, and psychological preparation. One-on-one support, peer mentoring, and supervised practice further enhanced parental confidence. In contrast, limited and inconsistent involvement in the standard care group, especially minimal paternal participation, may account for the observed differences. Discharge readiness is a critical component of the transition from hospital to home for families of preterm infants (27–29). It not only involves the acquisition of practical caregiving skills, but also the psychological preparedness and confidence required to assume full responsibility for infant care. It has been demonstrated that inadequate discharge preparation is related to the increased parental anxiety, reduced caregiving efficacy, and higher rates of unplanned healthcare utilization following NICU discharge (30–32). Mothers and fathers often report feeling overwhelmed by the sudden shift from a highly monitored environment to home-based care, particularly when they have had limited involvement during hospitalization (32). Interventions that provide structured education, hands-on practice opportunities, and emotional support, such as FICare, may enhance discharge readiness by promoting competence, reducing uncertainty, and promoting parental autonomy. Importantly, the inclusion of both parents in discharge planning is essential, as shared caregiving responsibility contributes to better family functioning and infant outcomes in the post-discharge period.

With respect to infant clinical outcomes, FICare was associated with significantly better nutritional parameters, including earlier initiation of enteral feeding, faster progression to full enteral feeds, earlier introduction of breast milk and direct breastfeeding, and higher discharge weight. These findings align with those of previous studies, indicating that parental presence and involvement may positively influence feeding outcomes and growth in preterm infants (33–35). The mechanism by which FICare improves nutritional outcomes is likely multifactorial. Active maternal participation enhances early and consistent breast milk expression, while timely skin-to-skin contact (kangaroo care) promotes thermoregulation, metabolic stability, and feeding tolerance (35). Moreover, repeated parental presence may enable better cue-based feeding and improve the coordination of infant suck-swallow-breathe reflexes, contributing to more efficient oral feeding development. Importantly, despite these outcomes, no significant differences were found in respiratory support duration (e.g., mechanical ventilation and CPAP) or length of NICU stay between the two groups, suggesting that the clinical safety and stability of infants were not compromised by the increased parental involvement. This finding challenges previously reported concerns that non-professional caregiver participation might interfere with clinical workflows or increase the risk of nosocomial complications (36, 37).

The mode of delivery can significantly impact maternal physical recovery, emotional well-being, and psychological outcomes. In the present study, 80.95% of mothers in the control group had a vaginal delivery, while 19.05% had a cesarean section. In the FICare group, 66.67% had a vaginal birth, and 33.33% underwent a cesarean section. Vaginal delivery is generally associated with quicker recovery, while cesarean sections involve longer recovery time, increased discomfort, and a higher risk of complications, leading to higher levels of stress, anxiety, and feelings of inadequacy. Mothers who undergo cesarean delivery may experience postnatal depression, anxiety, and trauma more frequently, partly due to a perceived loss of control during the birth process. This may also limit their early involvement in infant care, leading to feelings of detachment. In contrast, vaginal delivery typically promotes faster recovery and greater involvement in newborn care, promoting better psychological outcomes and discharge readiness.

It is important to consider that the Chinese cultural context may influence parental experiences and psychological outcomes in the NICU setting. In traditional Chinese culture, the family unit is highly valued, and parental roles are often more clearly defined, with a strong emphasis on the mother's responsibility for caregiving (31, 34). This cultural context may influence both the psychological distress parents experience and their readiness for discharge. For instance, mothers may feel an elevated sense of guilt or inadequacy if they are unable to care for their infant in a culturally prescribed manner, especially if their involvement is limited by NICU protocols (22). This can exacerbate feelings of stress and anxiety, particularly in the absence of comprehensive, family-centered care. Conversely, FICare's approach, which encourages active involvement from both parents, may alleviate these concerns by empowering mothers and fathers with the skills and knowledge they need to feel confident in their caregiving roles, thereby improving discharge readiness. In Chinese society, paternal involvement in caregiving has historically been more limited compared with Western cultures, where shared parenting responsibilities are more commonly practiced (29, 30). However, this study demonstrated that fathers in the FICare group experienced significant improvements in psychological outcomes and caregiving competence. These findings demonstrate that expanding paternal involvement in the NICU, as found in FICare, could have particular cultural significance in China by challenging traditional gender roles and promoting a more equitable distribution of caregiving responsibilities. The psychological benefits for fathers, including reductions in anxiety, stress, and trauma symptoms, may reflect a shift toward more active and shared parenting roles that align with evolving attitudes toward fatherhood in contemporary Chinese society. However, in the context of Chinese families, discharge readiness may also be influenced by the presence of extended family members. In China, it is common for grandparents or other relatives to play a significant role in childcare (21). Thus, while FICare programs that emphasize parental involvement are beneficial, they may need to consider the dynamics of extended family support systems. For example, the cultural expectation for maternal and paternal roles may be altered by the presence of grandparents or other family members in the caregiving process (26). This cultural factor may affect how parents assess their own readiness for discharge and caregiving confidence. It is important that FICare interventions in China integrate family-centered approaches that acknowledge and involve extended family members in the caregiving process, enhancing parental confidence and ensuring a smooth transition from hospital to home. In Chinese culture, the mode of delivery is often regarded as a significant event, and the mother's emotional response to it can have lasting effects on her sense of identity and motherhood (36). Understanding these cultural implications can help healthcare providers with more empathetic support and care for mothers, particularly those recovering from cesarean sections, ensuring that they feel supported in their caregiving role.

This study has several limitations that warrant consideration. Firstly, the study was conducted at a single tertiary NICU center, which might limit the generalizability of the findings to other institutions with different patient populations, staffing models, or resources. Secondly, the nature of the intervention precluded participant and provider blinding, increasing the risk of performance and detection bias, especially for subjective outcomes, such as psychological assessments. Thirdly, psychological outcomes were evaluated only up to three months postpartum; longer-term effects on parental mental health, caregiving confidence, and infant developmental outcomes remain unknown and warrant further longitudinal investigation. Additionally, both the control and FICare groups had relatively small sample sizes, which might limit the statistical power of the analyses and the ability to detect smaller effect sizes. The small sample size could also reduce the generalizability of the findings, as the results might not fully reflect the diversity of NICU populations across different settings or regions. Larger, multicenter studies are needed to confirm these findings and assess their applicability to broader populations. Furthermore, the questionnaires in this study were self-translated by the authors instead of using validated Chinese versions. Future research should use validated Chinese versions or employ a professional translation and validation process to ensure accuracy and cultural relevance.

## 5 Conclusion

This study demonstrated that FICare could significantly improve psychological outcomes and discharge readiness among parents of preterm infants in the NICU. Compared with standard care, FICare reduced symptoms of trauma, anxiety, depression, and stress in both mothers and fathers, while also enhancing caregiving competence and readiness for discharge. Infants in the FICare group achieved nutritional milestones earlier and had higher discharge weights, without prolonged NICU stays or increased respiratory support. These findings highlight the clinical and psychosocial value of FICare and support its broader implementation in NICU settings. Further multicenter, long-term studies are warranted to validate and expand these results.

## Data Availability

The original contributions presented in the study are included in the article/Supplementary Material, further inquiries can be directed to the corresponding author.
